# Eyes on the Mind: Investigating the Influence of Gaze Dynamics on the Perception of Others in Real-Time Social Interaction

**DOI:** 10.3389/fpsyg.2012.00537

**Published:** 2012-12-03

**Authors:** Ulrich J. Pfeiffer, Leonhard Schilbach, Mathis Jording, Bert Timmermans, Gary Bente, Kai Vogeley

**Affiliations:** ^1^Neuroimaging Group, Department of Psychiatry, University Hospital CologneCologne, Germany; ^2^Institute of Neuroscience and Medicine – Cognitive Neurology (INM3), Research Center JuelichJuelich, Germany; ^3^Max-Planck-Institute for Neurological ResearchCologne, Germany; ^4^Faculty of Human Sciences, Department of Media and Social Psychology, University of CologneCologne, Germany

**Keywords:** gaze-following, joint attention, shared attention, social interaction, agency, mentalizing, eye-tracking

## Abstract

Social gaze provides a window into the interests and intentions of others and allows us to actively point out our own. It enables us to engage in *triadic* interactions involving human actors and physical objects and to build an indispensable basis for coordinated action and collaborative efforts. The object-related aspect of gaze in combination with the fact that any motor act of looking encompasses both input and output of the minds involved makes this non-verbal cue system particularly interesting for research in embodied social cognition. Social gaze comprises several core components, such as gaze-following or gaze aversion. Gaze-following can result in situations of either “joint attention” or “shared attention.” The former describes situations in which the gaze-follower is aware of sharing a joint visual focus with the gazer. The latter refers to a situation in which gazer and gaze-follower focus on the same object *and* both are aware of their reciprocal awareness of this joint focus. Here, a novel interactive eye-tracking paradigm suited for studying triadic interactions was used to explore two aspects of social gaze. Experiments 1a and 1b assessed how the latency of another person’s gaze reactions (i.e., gaze-following or gaze version) affected participants’ sense of agency, which was measured by their experience of relatedness of these reactions. Results demonstrate that both timing and congruency of a gaze reaction as well as the other’s action options influence the sense of agency. Experiment 2 explored differences in gaze dynamics when participants were asked to establish either joint or shared attention. Findings indicate that establishing shared attention takes longer and requires a larger number of gaze shifts as compared to joint attention, which more closely seems to resemble simple visual detection. Taken together, novel insights into the sense of agency and the awareness of others in gaze-based interaction are provided.

## Introduction

The visual system is a major source of information about the environment. In face-to-face social encounters it is not only a source of information but also a crucial means of non-verbal communication. Imagine the following everyday situation: you are sitting at the bar of a pub gazing contemplatively at your empty glass. Suddenly the bartender walks by and observes that your eyes are directed at the empty glass. As soon as you direct your gaze at him and back to the glass he will – without words – understand that you need another drink. Such instances of “social gaze” demonstrate how meaning can be conveyed by simple acts of looking. A considerable amount of research has been devoted to the development and function of social gaze (Argyle and Cook, 1976; Mundy and Newell, 2007; Shepherd, 2010). Gaze represents a non-verbal cue system which reflects perception and action simultaneously, or in which, as Gibson and Pick, 1963, p. 368) have noted, “any act of looking can be treated as a source of stimulation as well as a type of response.” Its salience in social encounters makes gaze a perfect tool to study “online” social interaction, i.e., face-to-face interaction between two persons in real-time (Schilbach et al., 2011).

Mainly due to methodological constraints, the study of online interaction has largely been neglected by researchers in social cognition (Schilbach et al., in press). In recent years, however, there have been exciting advances to create tools for the investigation of non-verbal and especially gaze-based social interaction (Redcay et al., 2010; Wilms et al., 2010; Staudte and Crocker, 2011; Bayliss et al., 2012). For example, Redcay et al. (2010) established a setup in which participants inside an MRI scanner could either interact face-to-face with an experimenter via a live video feed or watch a recording of the experimenter’s behavior during previous interactions, thereby enabling the investigation of the processing of dynamic features of social interaction. Staudte and Crocker (2011) designed a series of experiments in which participants interacted with an artificial agent (i.e., a robot) in order to study the dynamic coupling between gaze and language in verbal human-robot interaction. Recently, Wilms et al. (2010) introduced an interactive eye-tracking setup which allows participants to interact with an anthropomorphic virtual character in a gaze-contingent manner. A similar program has been created recently by another group to study face-to-face interaction in social contexts (Grynszpan et al., 2012).

The advent of virtual reality techniques for research in neuroscience and psychology (Tarr and Warren, 2002; Bohil et al., 2011) has raised the general question why we need these displays to study human cognition. Bohil et al. (2011, p. 752) have noted that “an enduring tension exists between ecological validity and experimental control” in psychological research. They suggest that virtual reality techniques provide a way out of this dilemma because they provide naturalistic, real-world-like displays whilst offering full control over a selected set of experimental variables. Indeed, studies addressing the validity of using virtual characters have demonstrated that the interaction with virtual agents elicits social behaviors which are similar to real interaction (von der Pütten et al., 2010) and that uncontrolled aspects of another person’s outer appearance and non-verbal behavior can be filtered out while participants’ overall impression of an interaction remains intact (Vogeley and Bente, 2010). In addition, avatar- and video-mediated communication have shown to create comparable levels of experienced social presence and intimateness (Bente et al., 2008).

Before such paradigms can be used to study gaze in more complex social scenarios, basic parameters of different processes of social gaze need to be identified. Several of these processes have been defined by Emery (2000): *direct (or mutual) gaze* – a situation where two individuals direct their gaze at each other – is described as the most basic process of social gaze. If one individual detects that the other averts its gaze this can serve as a cue for a *gaze-following* reaction to the other’s novel focus of visual attention. This results in a situation of *joint attention (JA)*, in which the gaze-follower is aware that he and the gazer have the same focus of attention – for instance, an object in the environment. In other words, in JA another person’s gaze is hence used as a cue to this person’s visual attention. This has been argued to represent a crucial prerequisite for the gaze-follower to infer the gazer’s mental states (e.g., thoughts, intentions, feelings…) regarding an object of joint focus (Gopnik et al., 1994), an ability commonly referred to as mentalizing (Frith and Frith, 2006). Notably, JA does not require the gazer to be aware of the gaze-follower’s reaction. In contrast, *shared attention (SA)* requires that *both* individuals are aware of focusing on the same object *and* of each other’s reciprocal awareness of this joint attentional focus (Emery, 2000). Moreover, SA has been argued (Moll and Tomasello, 2007) to involve the gazer’s intention to direct the other’s gaze to a certain object in order to achieve a shared goal or share an experience, thereby providing a behaviorally accessible measure of shared intentionality. Notably, different but often overlapping descriptions of JA or SA exist in the literature (e.g., Clark, 1996; Povinelli and Eddy, 1996; Tomasello et al., 2005; Frischen et al., 2007; Mundy and Newell, 2007). The study presented in this article is largely guided by the comparably mechanistic account of Emery (2000), which provides a clear conceptual distinction between JA and SA that is suited to provide empirical access to these processes.

Joint and shared attention constitute so-called triadic social interactions. In contrast to dyadic interactions which develop early in infancy and involve processes such as mutual gaze or reciprocal emotional displays (Stern, 1974), triadic interactions are characterized by involving “the referential triangle of child, adult, and some third event or entity to which the participants share attention” (Carpenter et al., 1998, p. 1). The establishment of reference to a certain aspect of the environment in a triadic interaction thus creates a form of perceptual common ground (Clark, 1996). This is a prerequisite for understanding each other’s goals and intentions regarding the object of joint focus. So far, however, the temporal and spatial dynamics of gaze in triadic interactions have not been studied systematically using interactive (i.e., gaze-contingent) paradigms (for discussion, see Becchio et al., 2010; Schilbach et al., in press). Although pictures of objects have been used in gaze cueing studies (Bayliss et al., 2006, 2007; van der Weiden et al., 2010), interactive eye-tracking studies so far have been limited to simple geometric shapes as stimuli (Schilbach et al., 2010; Wilms et al., 2010; Pfeiffer et al., 2011).

Using pictures of real-world objects, the current study employs a more ecologically valid interactive eye-tracking setup to address the following questions: (1) *How does the perception of JA depend on the congruency (i.e., gaze-following and gaze aversion) and latency of another person’s gaze reactions?* In experiments 1a and 1b, the effect of the congruency of gaze reactions – gaze-following and gaze aversion – as well as the latency with which these reactions follow participants’ gaze shifts was manipulated. To this end, participants interacted with a virtual character in brief triadic interactions in which the character would either engage in joint or in non-joint attention (NJA) with different latencies. After each reaction, participants had to indicate how related they experienced this reaction to their own behavior. We argue that this can be taken as a measure to which *degree* participants experienced agency, i.e., that the other’s reaction is a consequence of their own action. In its prevalent definition, the sense of agency is described as an all-or-none phenomenon relating to the awareness that we are the initiators of our own actions (de Vignemont and Fourneret, 2004; Synofzik et al., 2008). However, the sense of agency also encompasses an awareness of the consequences (e.g., another person’s gaze shifts) inextricably linked to our actions (Bandura, 1989; Pacherie, 2012). As put forward by Pacherie (2012), in social interactions agency experience is not only influenced by high-level cognitive factors and sensorimotor cues, but also by perceptual consequences of one’s own actions, including the reactions of another person. Specifically, we hypothesize that participants experience gaze-following (which results in JA) as more strongly related to their own gaze behavior as compared to gaze aversion (which results in disparate attention). It is also predicted that the latency of gaze reactions modulates this experience: very short latencies, which might create an experience of coincidental looking, as well as very long latencies, which might disrupt the temporal contingency between actions, were supposed to decrease participants’ sense of agency. (2) *Does gaze behavior differ in situations of JA and SA*? Although the concepts of JA and SA are theoretically distinct, it has never been tested experimentally whether they correspond to differences in the dynamics of gaze behavior. In Experiment 2, participants engaged in a series of triadic interactions in which they were asked to indicate whenever they experienced JA or SA. We hypothesized that SA requires an increased number of gaze shifts and takes longer to establish as compared to JA.

## Materials and Methods

In this section, three different experiments will be described. These experiments largely rely on the same materials and methods. For the sake of brevity, those materials and methods that are common to all experiments will be indicated before the procedure of each experiment will be described separately.

### Participants

In sum, 95 healthy female and male persons aged 19–42 years (*M* = 25.86, SD = 6.23), with no record of neurologic or psychiatric illnesses volunteered for the study. The numbers for each individual experiment are given in the description of that particular experiment below. All participants were naïve to the scientific purpose of the study and were compensated for their participation (10 Euro/h). Prior to the experiment, participants were asked to sign a written consent form in which they approved that participation is voluntary and that data are used in an anonymized fashion for statistical analysis and scientific publication. The study followed the WMA Declaration of Helsinki (Ethical Principles for Medical Research Involving Human Subjects) and was presented to and approved by the ethics committee of the Medical Faculty of the University Hospital Cologne, Germany.

### Setup and materials

**Figure 1:**
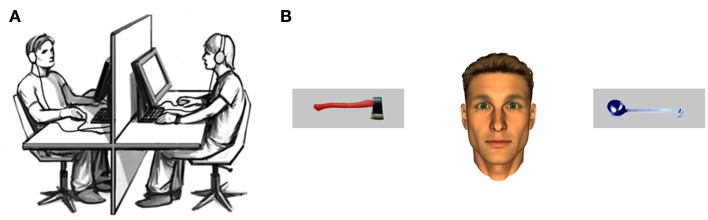
**(A)** Illustration of the interactive eye-tracking setup with the real participant on one side and the interaction partner – a confederate of the experimenter – on the other (taken from Pfeiffer et al., 2011, p. 2). **(B)** Example trial depicting the male anthropomorphic virtual character and pictures of two real-life objects.

We made use of an interactive eye-tracking program recently developed (Wilms et al., 2010). This method allows participants to interact with an anthropomorphic virtual character by means of their eye-movements. Using a high resolution eye-tracking device (Tobii^™^ T1750 Eye-Tracker, Tobii Technology AB, Sweden) with a digitization rate of 50 Hz and an accuracy of 0.5°, participants’ eye-movements could be detected exactly. Stimuli were presented on the 17″ TFT screen of the eye-tracker with screen resolution set to 1024 by 768 pixels. Both the participant and the confederate were seated at a distance of 80 cm from their respective eye-tracker as depicted in Figure 1A. The viewing angle subtended 32° × 24°. A PC with a dual-core processor and a GeForce 2 MX graphics board controlled the eye-tracker as well as stimulus presentation at a frame rate of 100 Hz. Integrated gaze extraction software (Clearview^™^, Tobii Technology AB, Sweden) made data available for real-time computation of stimulus presentation to the software package Presentation (Presentation^™^)^1^ which was used to control stimulus presentation in a gaze-contingent manner (for details on the algorithm see Wilms et al., 2010). All data were analyzed using PASW Statistics 20 (SPSS Inc., Chicago, IL, USA)^2^.

### Stimuli

One male and one female anthropomorphic virtual character were used in this study (Schilbach et al., 2010; Pfeiffer et al., 2011). Except for their eyes, the facial features of these characters were static in order to prevent the influence of non-verbal information other than gaze. Male participants interacted with the male character (exemplarily depicted in Figure 1B) and female participants with the female character, respectively. The potency of virtual characters to elicit social presence and the advantages of their usage in experiments on social cognition has been demonstrated previously (for detailed discussion, see Loomis et al., 1999; Bailenson et al., 2003; Vogeley and Bente, 2010).

The 32 object stimuli used here were taken from a previously published study (Bayliss et al., 2006) and consist of two different categories of everyday-life objects, i.e., typical “kitchen” and “garage” objects (Figure 1B). They were standardized with respect to likeability (*M* = 4.75, SD = 0.97 on a nine-level scale) and to participants’ ability to assign them to their respective category (accuracy *M* = 95.3%, SD = 2.66). Each of the objects was used in two different colors (blue and red) and was mirrored to create two different orientations (i.e., the handle pointing to the left or the right). They were presented within a gray rectangle with a size of 306 × 108 pixels. All pictures were analyzed with respect to their size and their luminescence to ensure physical consistency. The manipulations of color and orientation yielded a total of 128 different pictures, which allowed for the presentation of two new pictures in each trial. Figure 1B depicts an example of a stimulus screen.

### Cover story

Participants were led to believe that they would engage in a gaze-based interaction task with another participant and that the interaction would not be vis-à-vis but via virtual characters serving as avatars of their gaze behavior. More specifically, participants were instructed that their eye-movements would be conferred to a virtual character displayed on the screen of their interaction partner. Likewise, the eye-movements of their interaction partner would be visualized by a virtual character displayed on their screen. In fact, however, the interaction partner was a confederate of the experimenter and the virtual character’s eye-movements were always controlled by a computer program to ensure full experimental control. Participants were debriefed about this manipulation after the experiment and belief in the cover story was controlled during a post-experiment interview.

### Procedure

In the beginning of each experiment the participant and the confederate were seated in front of two eye-tracking devices. Female participants interacted with a female confederate, and male participants with a male confederate, respectively. Subsequently, they received written instructions on the computer screen. A room-divider visually separated both persons. After both of them indicated that they had understood the instructions, the participant’s eye-tracker was calibrated. To sustain the cover story, the experimenter pretended to be calibrating the eye-tracker of the interaction partner as well. In addition, during the experiment both persons were asked to wear ear protection so that the participant was not distracted from the task and to make verbal communication impossible.

### Experiment 1a

The first experiment aimed at assessing at which latencies participants experienced gaze reactions – either gaze-following or gaze aversion – of another person as contingent on their own gaze shifts. It consisted of two main conditions: (1) JA trials in which the virtual character followed the participant’s gaze and (2) NJA trials in which the virtual character did not follow the participant’s gaze but shifted its gaze toward the other object. In both conditions the latency of the virtual character’s gaze reactions was varied from 0 to 4000 ms in steps of 400 ms. This yielded eleven sub-conditions which were repeated eight times throughout the experiment, thereby resulting in a total of 176 trials which were presented in a randomized fashion.

Each trial started with an initiation phase in which participants were instructed to fixate the virtual character. Upon fixation two objects appeared to the left and the right of the virtual character. Participants were asked to shift their gaze to one of these objects as quickly as possible and to wait for the reaction of the virtual character. After the character’s gaze reaction the scene remained static for another 500 ms before participants had to indicate by button press how strongly related they experienced the gaze reaction of the other to their own gaze shift on a four-item scale (very related – rather related – rather unrelated – very unrelated). Each trial was followed by a short break in which a fixation cross was presented with a latency jittered between 1000 and 2000 ms. The total duration of the experiment was about 25 min.

In this experiment, 30 volunteers participated, out of which 27 (Mean age = 27.63, SD = 6.29, 15 female/12 male) entered the analysis. Two had to be excluded from data analysis because of technical problems and another one due to disbelief in the cover story.

### Experiment 1b

In order to enhance participants’ sensitivity to the timing of *gaze-following*, Experiment 1a was repeated without the non-JA condition, that is, the virtual character followed participants’ gaze in *all* trials. Participants were instructed that their putative interaction partner was instructed to always look at the same object. As each sub-condition (i.e., reaction latencies from 0 to 4000 ms in steps of 400 ms) was repeated 16 instead of eight times, Experiment 1b did not differ structurally from Experiment 1a.

There were 24 participants in this experiment. Only 21 (Mean age = 23.86, SD = 5.74, 14 female/7 male) were included in the analysis as two had to be excluded due to technical problems and one due to disbelief in the cover story.

### Experiment 2

The aim of this experiment was to assess whether the theoretically proposed processes of JA and SA differ with respect to the interaction dynamics. The experimental design contained a between-subject and a within-subject factor. The within-subject factor was the order of initiation of the interaction sequence (self-initiated vs. other-initiated) and the between-subject factor was task instruction (JA vs. SA). Prior to the experiment, participants were assigned in a randomized but gender-balanced fashion to either a JA or a SA group. In the JA group, participants were instructed to press a response button as soon as *they themselves were aware that both they and their interaction partner directed their attention to the same object*. In the SA condition, participants were asked to press the button as soon as *they were convinced that both of them were aware of each other directing their attention to the same object*. Particular caution was exerted to avoid any explanation that went beyond the descriptions written in italics above and any cues toward the theoretical concepts of JA and SA or related psychological processes.

In both JA and SA groups, the order of initiation of the interaction sequence (i.e., the within-subject factor) was manipulated block-wise. The initiator of a trial is the person who is the first to fixate one of the two objects on the screen. Participants either started with the self-initiated block in the first half of the experiment and then proceeded in the other-initiated block in the second half or vice versa. To avoid sequence effects, participants started with the self- or other-initiated block in an alternating fashion. Each block consisted of 32 trials. In the beginning of each trial two objects were shown for 3000 ms on the left and the right side of the screen so that participants could become acquainted to them and subsequently concentrate on the interaction task. After the acquaintance period the virtual character appeared in the center of the screen. This served as a cue to the initiation of the interaction. Participants were instructed that the establishment of mutual gaze with the virtual character was a prerequisite for the interaction sequence to start. Depending on the experimental block, there were two ways the interaction period could be initiated. (1) In trials of the self-initiated block participants were told to choose one object by fixating it and the virtual character followed their gaze. (2) In contrast, in trials of the other-initiated block the virtual character commenced the interaction by shifting its gaze to one of the objects. Participants were instructed to follow its gaze. As soon as the first gaze fixation on the virtual character (in the self-initiated condition) or on the chosen object (in the other-initiated condition) was detected, the dynamic interaction period started. When the participant looked at the virtual character, it responded by shifting its gaze to the participant to establish eye contact. When the participant looked back at the object, the virtual character followed his or her gaze. Gaze reactions of the virtual character followed with a latency that was jittered between 400 and 800 ms (i.e., latencies experienced as “natural” for human gaze reactions according to Experiments 1a and 1b). This interaction continued until participants – depending on the group they had been assigned to – indicated the experience of JA or SA (as described above) by pressing a button and thereby ending the current trial.

Overall, 43 participants participated in the study. As three of them were excluded due to technical problems, only 40 of them (Mean age = 24.75, SD = 5.15, 20 female/20 male) were included in the analysis.

## Results

### Experiment 1a

**Figure 2:**
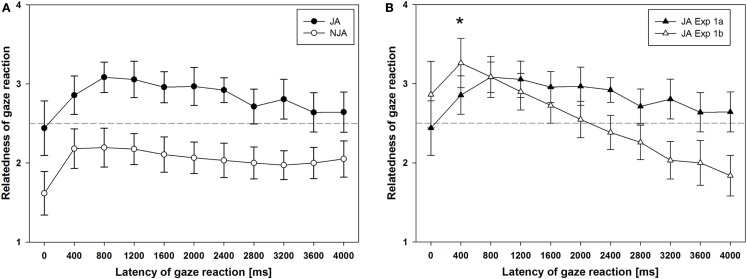
**(A)** The results from Experiment 1a, in which the interaction partner could either follow the gaze of the participant to engage in joint attention (JA) or avert his/her gaze to the other object to engage in non-joint attention (NJA). **(B)** In Experiment 1b the interaction partner always engaged in JA, only the latency of the gaze reaction is varied. For better comparability, the joint attention data of Experiment 1a (JA in the context of NJA as another option to act) are plotted together with the data from Experiment 1b (JA only).

The ratings of relatedness of the avatar’s gaze reactions are depicted in Figure 2A. A two-way ANOVA for repeated-measures with the factors gaze reaction (joint vs. non-joint) and latency (0–4000 ms in steps of 400 ms) showed a main effect of gaze reaction: as expected, gaze-following reactions resulting in JA were experienced as more related to participants’ gaze shifts as compared to gaze aversion resulting in NJA, *F*(1, 26) = 67.09, *p* < 0.001. In addition, there was a main effect of latency on participants’ ratings of relatedness, *F*(5.83, 92.54) = 5.38, *p* = 0.001 (Greenhouse–Geisser corrected, ε = 0.36, due to a violation of the assumption of sphericity). For both joint and NJA trials, participants rated immediate reactions with a latency of 0 ms as considerably less related to their own gaze shift than reactions with higher latencies. In addition, ratings of relatedness seemed to decrease linearly for latencies greater than 800 ms (see also the “Combined Analysis of Gaze-Following in Experiments 1a and 1b” below). There was no significant interaction between these two factors, *F*(6.3, 163.76) = 1.26, *p* = 0.28.

### Experiment 1b

Figure 2B shows the ratings of relatedness of the avatar’s gaze reaction to participants’ own gaze shift as a function of the latency of the reaction. A one-way repeated-measures ANOVA revealed that, similar to the results of Experiment 1a, there was a main effect of latency on participants’ rating of relatedness of the other’s gaze reaction, *F*(17.07, 54.87) = 26.78, *p* < 0.001 (Greenhouse–Geisser corrected, ε = 0.27). This effect was described by a highly significant linear trend, *F*(1, 20) = 53.14, *p* < 0.001, indicating a continuous decrease of relatedness ratings with increasing latency of gaze reactions.

### Combined analysis of gaze-following in Experiments 1a and 1b

In a separate set of analyses, we focused only on JA and compared the JA trials from Experiment 1a to Experiment 1b. The crucial difference between these two experiments was that in Experiment 1a the putative interaction partner had an additional option to react and could also avert his/her gaze, whereas in Experiment 1b the virtual character would always follow participants’ gaze, which participants were informed of during the instruction. In order to assess the influence of a second option to react on the perception of latency of gaze-following, we conducted a two-way repeated-measures ANOVA including only the JA trials from Experiment 1a and all trials from Experiment 1b with experiment as a between-subjects factor. There was a significant interaction between the factors experiment and relatedness rating, *F*(4.27, 196.3) = 11.02, *p* < 0.001 (Greenhouse–Geisser corrected, ε = 0.43). As Figure 2B shows, ratings from Experiment 1b (open circles), which consisted only of JA trials, suggest that participants experience gaze-following reactions as most related to their own gaze shift when they follow with a latency of 400 ms (*M* = 3.26, SD = 0.68). In Experiment 1a (filled circles) ratings for gaze reactions with a latency of 400 ms were significantly lower (*M* = 2.86, SD = 0.61), as shown by a *t*-test for independent samples, *t*(46) = −2.16, *p* = 0.038. Here, visual inspection of data suggests that maximum relatedness ratings were not reached before 800 ms. Furthermore, in Experiment 1b there was a continuous linear decrease of relatedness ratings beginning at 400 ms. This was confirmed by a highly significant linear trend, *F*(16.06, 42.67) = 53.14, *p* < 0.001, which is absent in the data of Experiment 1a, *F*(0.47, 17.49) = 0.7, *p* = 0.41. Taken together, these results suggest that when the interaction partner has no other choice but following participants’ gaze, relatedness ratings peak earlier as compared to a context in which the other can either react by gaze-following or by gaze aversion. In addition, participants’ are less sensitive to the latency of gaze-following in the context of action alternatives.

### Experiment 2

**Figure 3:**
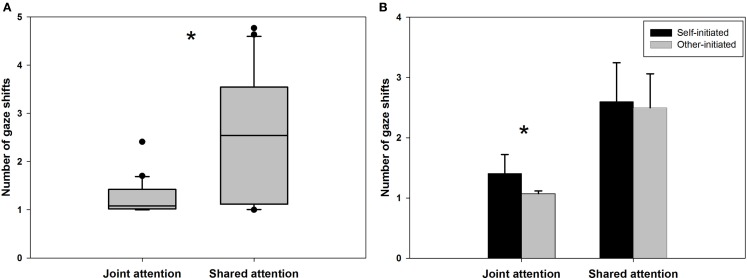
**(A)** A box plot illustrates the inter-individual variance of the number of gaze shifts before indicating the experience of joint as compared to shared attention. **(B)** Whether participants initiated the gaze-based interaction only affected the number of gaze shifts required to report a state of joint, but not shared attention.

An independent samples t-test indicated that significantly more gaze shifts were required to reach a situation of shared (*M* = 2.55, SD = 1.26) as compared to JA (*M* = 1.23, SD = 0.35). Furthermore, standard deviations indicate that the inter-individual variance was much higher in SA. This between-subject variance is also depicted in the box plot in Figure 3A. Importantly, the establishment of mutual gaze was a prerequisite for the initiation of the interaction to ensure that scan paths always began with a fixation of the virtual character. The increased number of gaze shifts also resulted in significantly longer trial durations in shared (*M* = 3886.39 ms, SD = 1838.91 ms) vs. JA (*M* = 2040.11 ms, SD = 974.64 ms), *t*(28.89) = −3.97, *p* < 0.001, *r* = −0.58. Interestingly, in JA participants showed significantly more gaze shifts in self-initiated trials (*M* = 1.41, SD = 0.68) compared to other-initiated trials (*M* = 1.07, SD = 0.10), *t*(19.79) = 2.18, *p* = 0.042, *r* = 0.33, while there was no such effect of initiation in SA, *t*(38) = 0.24, *p* = 0.81 (see Figure 3B), indicating that only the gaze dynamics of JA were influenced by the initiation of the interaction.

## Discussion

The present study introduced a novel interactive eye-tracking paradigm suitable to study multiple facets of triadic interactions between two agents and real-world objects in real-time. On a methodological level, this provides an important complement to previous work by our group which has not involved real objects but rather concentrated on the dyadic aspects of gaze-following and JA (Schilbach et al., 2010; Wilms et al., 2010; Pfeiffer et al., 2011). This methodological advancement was used for the empirical investigation of temporal and dynamic aspects of social gaze as a socially salient form of embodied actions with great ecological validity. In Experiments 1a and 1b, participant’s sense of agency was measured as a function of both the congruency and latency of another person’s gaze reaction. In Experiment 2, differences in gaze dynamics and trial duration resulting in JA and SA were examined. These results provide interesting insights into gaze behavior and the experience of gaze reactions in an ecologically valid but experimentally controllable setting. Conceptual as well as methodological implications are discussed in the following.

### Effects of the congruency of gaze reactions

Experiments 1a and 1b investigated how related participants experienced different latencies of gaze reactions to their own gaze behavior by varying these latencies and the congruency of reactions (i.e., gaze-following vs. gaze aversion) systematically. In the following, we suggest that the experience of relatedness can be taken as a measure of the sense of agency (Pacherie, 2012).

It was first predicted that the congruency of the other’s gaze reaction (gaze-following vs. gaze aversion) strongly influences participants’ sense of agency, as measured by their experience of relatedness. Indeed, results indicated that gaze-following is experienced more strongly related to one’s own gaze shifts as compared to gaze aversion. It is highly plausible that this relates to a positive valence that has been associated with gaze-following in comparison to gaze aversion. The literature provides indirect evidence for positive and negative evaluations of gaze-following and gaze aversion, respectively. In a recent study aiming at unraveling the expectations of participants’ regarding the behavior of a human interaction partner, we asked participants to interact with a virtual character in a similar interactive eye-tracking setup as in the present study (Pfeiffer et al., 2011). In order to distinguish social from non-social interaction, participants were led to believe that in any given interaction block consisting of a number of gaze trials the virtual character could either be controlled by another person or a computer algorithm. Their task was to decide based on the virtual character’s gaze reactions whether they had been interacting with a human or a computer. Unbeknownst to participants, the reactions were always controlled by a computer algorithm to allow full experimental control. Results demonstrated that the proportion of human ratings increased linearly with increasing numbers of gaze-following trials in an interaction block, thereby indicating that in such simple gaze-based interactions, gaze-following and JA are taken as most indicative of true social interaction. This supports the present finding that gaze-following results in an enhanced experience of agency as expressed by higher ratings of self-relatedness.

Another set of studies emphasizes the positive valence of gaze-following in contrast to gaze aversion. A recent study used interactive eye-tracking in an MRI scanner to compare other- and self-initiated situations of JA and NJA and demonstrated a specifically positive valence of self-initiated JA (Schilbach et al., 2010). Results indicated that self-initiated JA correlates with activity in the ventral striatum, a brain region which is a part of the brain’s reward system and whose activation has been linked to hedonic experiences (Liu et al., 2007). There is also evidence for negative affective evaluations of gaze aversion. For example, Hietanen et al. (2008) showed in an EEG study that watching pictures of persons averting their gaze leads to avoidance-related neural activity, whereas watching pictures of persons with direct gaze correlated with approach-related signals. Furthermore, persons who avert their gaze are judged as less likeable and attractive as compared to persons exhibiting direct gaze (Mason et al., 2005) and gaze aversion is understood as a non-verbal cue to lying and insincerity (Einav and Hood, 2008; Williams et al., 2009). It is conceivable that the intrinsically rewarding nature of initiating social interaction by leading someone’s gaze in combination with the implicitly negative evaluation of averted gaze plays a prominent role in the increased feeling of relatedness for gaze-following as compared to gaze aversion.

### The influence of reaction latencies and action possibilities on the experience of gaze reactions

We hypothesized that, while very short latencies might be perceived as coincidental, reactions with long latencies might be experienced as non-contingent upon one’s own behavior. Indeed, the most obvious finding was that in all conditions reactions with a latency of 0 ms were experienced as considerably less related than the subsequent latency levels of 400 and 800 ms. This result is plausibly explained by the fact that a certain minimal delay needs to be present until a reaction can be experienced as causally linked to (or launched by) any given preceding action and not just as mere coincidence (Scholl and Tremoulet, 2000). Literature suggests that the natural latency of normal saccades (i.e., not express saccades) to any form of visual displacement on a screen is between 200 and 250 ms (Saslow, 1967; Yang et al., 2002). Although our results do not precisely show at which latencies a reaction is experienced as merely coincidental, it is conceivable that saccadic latencies are implicitly taken into account in participants’ ratings of relatedness and that gaze reactions with latencies below 250 ms are therefore considered unrelated. However, further experiments are needed to investigate in detail how latencies of gaze reactions between 0 and 400 ms are experienced.

Notably, however, the experience of different latencies of a gaze-following reaction appears to depend on the other person’s options to act. When the other person can choose to follow or to avert her eyes, there is hardly any effect of latency on the experience of relatedness and even reactions with a substantial delay of 4000 ms are experienced as rather related. In contrast, when the other person always engages in gaze-following relatedness ratings decrease linearly starting at a latency of 400 ms. Furthermore, reactions with latencies of more than 2000 ms are experienced as unrelated to one’s own gaze shifts – they fall below the dashed line symbolizing a neutral rating in Figure 2B, and thereby reach the level of unrelatedness that is associated with NJA.

The effect of the other person’s options for action is interesting in that it throws new light on the role of perceived causality for one’s sense of agency, which traditionally has to do with predicting the sensory consequences (avatar gaze shift) of self-produced actions (own gaze shift). This means that in a joint context, whereas my sensorimotor cues with respect to my own action remain identical to non-joint situations, I perceive the consequences of my actions *in the actions of the other person*. Therefore, the nature of the other person’s behavior will have a bearing on my experience of self-agency. In particular, as Pacherie (2012) notes, the strength of the sense of agency is related to how well our predictions regarding another person’s reaction to our own actions match with the actual reaction. This is specifically true in small-scale interactions – as in our experiments – in which every aspect of the interactors’ behavior is accessible. Rather than investigating sense of agency in an all-or-none fashion, we therefore interpreted participants’ ratings of relatedness of the other’s gaze reaction as a measure of how strongly they experienced agency in a given gaze trial.

Adopting this view of agency, the results of experiments 1a and 1b could reflect the role of perceived causality for one’s sense of agency. Haggard et al. (2002) have suggested that sense of agency depends crucially on the intentionality of the agent and found that it decreases with increasing action-outcome delays, as it does in Experiment 1b, and to a lesser degree in Experiment 1a. Subsequent research has shown that not only intentionality, but also *perceived* causality is crucial for the sense of agency. Buehner and Humphreys (2009) found that, when keeping action-outcome constant, given a strong perceived causal link, intentional binding was preserved at action – outcome delays of up to 4 s, as in Experiment 1a. However, there is a less persistent sense of agency in Experiment 1b although the actual causal link is stronger due to the avatar always following my gaze. This could mean that perceived causality is less important for my sense of agency in an interactive context. More plausibly, it could be that in an interactive context, since I am dealing with another agent, the evaluation of my own actions as causally efficacious is only meaningful *when I know that the other has different options for action*. Put otherwise, if I have to evaluate my own sense of agency, *given* that the effect is observed in the behavior of *another* agent, my judgment could be influenced crucially by the sense of agency I am able to attribute to the other (as suggested in Schilbach et al., in press). Further research is needed to look at the interdependency of one’s sense of agency for self and other in interaction, but the data from the first experiment show that there is a difference between how sense of agency is experienced in social as compared to non-social situations.

### Differences in gaze dynamics between joint and shared attention

In Experiment 2, the dynamics of gaze behavior in situations of JA and SA were assessed while making use of the temporal parameters uncovered in Experiment 1b. As described in the introduction, the necessary criteria for *joint attention* require only one of the interaction partners to be aware of the joint focus of attention. *Shared attention*, however, warrants *both* gazer and gaze-follower to be simultaneously aware of focusing on the same object *and* on each other’s awareness of focusing on the same object (Emery, 2000). Results clearly indicate that participants required a significantly higher number of gaze shifts between objects and the virtual character in order to establish SA as compared to JA. As a consequence of this, trial length was considerably longer. JA required only slightly more than one gaze shift on average and is reached significantly earlier in self- vs. other-initiated trials. This indicates that participants were able to make inferences about the emergence of JA by focusing on the object and seemingly observing their partner’s gaze reaction at the same time. Due to the impossibility of fixating two spatially separated objects simultaneously, these data demonstrate that a peripheral and quick recognition of the other’s gaze reaction is sufficient for the establishment of JA. In contrast to SA, the establishment of JA happens rapidly and is characterized by considerably less inter-individual invariance (see Figure 3A). This suggests that JA is characterized by the mere detection of the other’s focus of attention, thereby possibly representing a visual detection task rather than a mentalizing task. Unfortunately, it is not directly possible to compare reaction times between the present results and findings on visual detection. Previous studies have not used interactive settings but concentrated on the detection of objects in real-world scenes (Biederman, 1972) or on the detection of gaze direction in static displays (Franck et al., 1998). Using interactive eye-tracking, however, the link between JA and visual detection could now be assessed specifically.

In contrast, such an observation of the other’s gaze behavior “out of the corner of the eyes” appears to be insufficient for a reliable identification of a situation of SA. It has previously been argued that SA might be characterized by an increased level of interactivity (Staudte and Crocker, 2011). According to Kaplan and Hafner (2006), true SA requires a monitoring and understanding of the intentions of the other in a coordinated interaction process and is only reached when “both agents are aware of this coordination of “perspectives” toward the world” (Kaplan and Hafner, 2006, p. 145). The increased number of gaze shifts between the virtual character’s face and the object and the correlated increase in trial length are indicative of such a coordinated interaction aimed at an alignment of intentions. Determining whether another person is aware of the object jointly focused upon as well as of “us” being aware of us being aware requires thinking about the other’s mental states. This is reflected by the dynamics of gaze behavior which exceed the simple detection of a gaze shift to a joint focus of attention. In the vast majority of trials in the JA condition there is not a single look back to the virtual character’s face, while this is practically always the case in the SA condition (Figure 3): participants have to re-establish eye contact at least once before they indicate to experience SA. It has recently also been shown in an interaction task within a minimalist virtual environment that higher complexity and reciprocity in the dynamics of a *tactile* interaction leads to the experience of interacting with another human agent (Auvray et al., 2009). The experience of non-verbal social interaction therefore more generally seems to hinge upon certain elaborate dynamics between actions and reactions.

A final observation refers to the substantial inter-individual variance in the number of gaze shifts participants exhibit before indicating the experience of SA (cf. Figure 3A). This connotes that gaze behavior as an embodied correlate of mentalizing is subject to greater inter-individual differences as compared to gaze behavior in a visual detection task. Literature suggests that inter-individual differences in personality traits and behavioral dispositions strongly influence the performance in different types of mentalizing tasks, i.e., tasks that require reasoning about other persons’ mental states. For example, self-reported measures of empathy (Baron-Cohen and Wheelwright, 2004) or of the drive to do things systematically (i.e., systemizing, Baron-Cohen et al., 2003) as well as the personality trait of agreeableness (for a detailed discussion, see Nettle and Liddle, 2008) have been shown to affect mentalizing in a variety of tasks. More studies are required in order to determine which personality traits or behavioral dispositions result in the observed variance of gaze patterns in SA.

Taken together, the findings reported in this paper can be taken as a first fine-grained description of the temporal and spatial dynamics of social gaze in triadic interactions and their influence on our sense of agency and awareness of the mental states of others. Further assessment of the underlying mental processes is required to understand how manipulations of these aspects change our experience of a social interaction and our perception of the interaction partner.

### Outlook

Interactive eye-tracking paradigms incorporating virtual characters have proven specifically useful for the study of social interaction face-to-face and in real-time (Schilbach et al., in press). One major asset of such studies is that the results can be immediately fed back into novel designs with even greater ecological validity. This can stimulate the development for therapeutic tools to learn or improve non-verbal communication in autism spectrum disorders. These are characterized by impairments of the ability to interact with others, as well as by a specific deficiency in reading information from the eye region and interpreting gaze cues (Senju and Johnson, 2009). For example, autistic persons have problems engaging in JA – this is most apparent for the initiation of JA, although responding to another person’s bid for JA can also be problematic (Mundy and Newell, 2007). In a recent report on attempts to teach autistic children to initiate and respond to bids of JA, they were required to engage in triadic interactions with an instructor and different kinds of toys (Taylor and Hoch, 2008). As this setting made eye contact difficult, JA was initiated by the instructor by pointing at an object instead of gazing at it. In the condition in which the children were supposed to initiate JA, they were prompted verbally to do so and explicitly told how to do it. A gaze-contingent display would be advantageous here for several reasons: first of all, the interaction with an avatar would be less distressing for autistic persons than real social interaction. Especially in the beginning of a training program this might be beneficial. Secondly, the training program could be designed in a highly structured manner. Features of the avatar’s gaze behavior such as timing, gaze direction, or the length of direct gaze could be varied systematically while other facial features can be kept constant in order to prevent sensory overload. Thirdly, the simultaneous recording of eye-movements can be used to analyze scan paths in order to detect difficulties or peculiarities in the participant’s gaze behavior. Furthermore, using interactive eye-tracking allows changing the avatar’s reactions depending on the participant’s gaze behavior in real-time. Lastly, a virtual setting provides more options to highlight and manipulate objects, prompt certain actions, or deliver reinforcement for correct behavior.

Very recently, first attempts have been made to design gaze-contingent virtual reality applications (Bellani et al., 2011; Lahiri et al., 2011). Lahiri et al. (2011) designed a virtual reality application for autistic adolescents in which they are required to interact with a realistically designed virtual classmate. Their task was to make this classmate as comfortable as possible by their behavior. They were positively reinforced the more they looked at the eyes of the character or followed their movements to an object on the screen. A gaze-contingent algorithm inspired by the one invented by Wilms et al. (2010) was used to detect fixations within predefined regions of interest (i.e., eyes, face, object) and to determine the kind of reinforcement depending on when and how long these regions were fixated. This provides a very interesting example for an implicit training of non-verbal social skills using a gaze-sensitive virtual environment. Although this approach is promising, therapeutic tools still have difficulties providing the avatars with realistic gaze behavior (Bellani et al., 2011). Although clearly more work is needed, results from the present study could potentially be incorporated into virtual therapeutic tools.

## Conclusion

A thorough exploration and understanding of the parameters of social gaze is crucial for the investigation and understanding of social interactions in gaze-contingent paradigms (Wilms et al., 2010; Bayliss et al., 2012; Grynszpan et al., 2012) and for the formulation of hypotheses regarding people’s gaze behavior in online interaction (Neider et al., 2010; Dale et al., 2011). In addition, recent advances have been made to the development of dual eye-tracking setups which allow for investigating the gaze behavior of two participants interacting and collaborating in a shared virtual environment (Carletta et al., 2010). Although this approach is very promising, the design of tasks allowing for an assessment of interaction dynamics while controlling variables affecting the interaction still remains a challenge. Before true interaction without simulated others can be investigated, the use of interactive eye-tracking paradigms provides an important tool to study social gaze behavior in persons who experience being engaged and being responded to in an interaction.

## Conflict of Interest Statement

The authors declare that the research was conducted in the absence of any commercial or financial relationships that could be construed as a potential conflict of interest.

## Footnotes

^1^http://www.neurobs.com

^2^www.spss.com
